# Berberine inhibits the expression of hypoxia induction factor-1alpha and increases the radiosensitivity of prostate cancer

**DOI:** 10.1186/1746-1596-9-98

**Published:** 2014-05-27

**Authors:** Qu Zhang, Chi Zhang, Xi Yang, Baixia Yang, Jinfeng Wang, Yahui Kang, Zhongming Wang, Defan Li, Guanhong Huang, Zhaoming Ma, Xinchen Sun, Jing Cai, Guangzhou Tao, Shengbin Dai, Weidong Mao, Jianxin Ma

**Affiliations:** 1 Department of Radiotherapy, The Second People’s Hospital of LianYungang, Lianyungang Hospital Affiliated to Bengbu Medical College, Xingfu Road 161, Lianyungang, Jiangsu Province 222000, China; 2 Department of Radiotherapy, The First Affiliated Hospital of Nanjing Medical University, Nanjing, Jiangsu Province, China; 3 Department of Radiotherapy, Nantong Tumor Hospital Affiliated to Nantong University, Nantong, Jiangsu Province, China; 4 Department of Radiotherapy, The First People’s Hospital of Huaian, Huaian, Jiangsu Province, China; 5 Department of Radiotherapy, Taizhou People’s Hospital, Taizhou, Jiangsu Province, China; 6 Department of Radiotherapy, Jiangyin People’s Hospital, Wuxi, Jiangsu Province, China

**Keywords:** Berberine, Radiosensitivity, Prostate cancer, HIF-1α

## Abstract

**Abstract:**

**Background**

The radiation resistance of prostate cancer remains the primary obstacle to improve patient survival. This study aimed to investigate the effects of berberine, a commonly used natural product, on the radiosensitivity of prostate cancer.

**Methods**

Prostate cancer cell line LNCaP and DU-145 were subjected to hypoxia and/or ionizing radiation (IR), in the presence or absence of berberine treatment. Cell growth and colony formation, and apoptosis were evaluated. Moreover, LNCaP cells were xenografted into nude mice and subjected to IR and/or berberine treatment. The expression of HIF-1α and VEGF in prostate cancer cells and xenografts was detected by Western blot analysis.

**Results**

Berberine increased radiosensitivity of prostate cancer cells and xenografts in a dose dependent manner, and this was correlated with the inhibition of HIF-1α and VEGF expression.

**Conclusions**

Berberine may inhibit the expression of HIF-1α and VEGF and thus confer radiosensitivity on prostatic cancer cells. Berberine has potential application as an adjuvant in radiotherapy of prostatic cancer.

**Virtual Slides:**

The virtual slide(s) for this article can be found here: http://www.diagnosticpathology.diagnomx.eu/vs/1519827543125021.

## Background

Prostate cancer (PCa) is the most common malignancy in men and the second leading cause of death from cancer. Asian prostate cancer patients had better survival but the incidence rate of PCa has been increasing rapidly in recent years [1]. Currently, radiation therapy is the main treatment for PCa and provides excellent local control and increased overall survival for PCa patients [2]. However, some PCa patients exhibit radiation resistance and develop metastatic disease in less than 5 years [3]. Thus it is an important issue to reduce radiation resistance and increase radiosensitivity of PCa.

Hypoxia is a common feature of malignant tumors and makes solid tumors resistant to ionizing radiation (IR) [4]. Hypoxia inducible factor-1 (HIF-1) is one of the main regulators of cell response to hypoxia. HIF-1α is a subunit of HIF-1 and regulates the expression of a variety of downstream target genes, including vascular endothelial growth factor (VEGF), and could confer resistance to radiation therapy and chemotherapy [5,6].

Berberine is a quaternary ammonium salt from the protoberberine group of isoquinoline alkaloids. A number of studies have shown that berberine has anti-tumor activity for a variety of cancers. However, the effects of berberine on PCa have not been reported. In this study, we showed that berberine could inhibit HIF-1α expression in hypoxic PCa cells and thus sensitize the cells to IR. Moreover, we found that berberine could sensitize nude mice bearing PCa cells to IR by inhibiting the expression of HIF-1α and VEGF.

## Methods

### Cell culture and treatment

Human prostate cancer cell lines LNCaP and DU-145 were purchased from Shanghai Cell Bank, and cultured in RPMI-1640 medium (Invitrogen) supplemented with 10% fetal bovine serum (Invitrogen), 100 U/ml penicillin and 100 mg/ml streptomycin at 37°C in 5% CO_2_ and 21% O_2_ or 0.8% O_2_ in a humidified atmosphere. Exponentially growing cells were detached using 0.05% trypsin-EDTA every 2–3 days. The cells were irradiated in ambient air with 6 MV X-rays at a dose rate of 5.66 Gy/min at room temperature. Berberine hydrochloride (>95%) was purchased from Sigma Aldrich.

### MTT assay

Cells in early log phase were trypsinized and plated in 96-well plates at a density of 4,000 cells/well. After 24 h, the medium was removed and replaced with fresh medium supplemented with different concentrations of berberine (20, 50, 100, 150, 200, 250, 300, 400 μM). Cell viability was detected on day 1, 2 and 3 by MTT assay following the manufacturer’s instructions. Briefly, 10 μl of MTT was added into each well to a final concentration of 0.5 mg/ml. After incubation for 4 h, the cell supernatants were removed and DMSO (150 μl) was added to dissolve MTT crystals (formazan). The absorbance of the samples at 490 nm was read using a Bio-Rad microplate reader (model 630; Hercules, CA, USA).

### Clonogenic assay

Cells in early log phase were trypsinized and plated in 6-well plates. Then the cells were treated with or without bererine for the indicated time and then subjected to X-rays of 2, 4, 6, 8 Gy at room temperature. The cells were incubated at 37°C for 12 days for LNCaP cells and 14 days for DU-145 cells, fixed with methanol and stained with Giemsa. Finally, the plates were inspected by microscopy and the number of the colonies with at least 50 cells was counted.

### Flow cytometry analysis of apoptosis

Cells in early log phase were trypsinized and plated in 6-well plates at a specific density. The cells were treated with or without bererine in normxia or hypoxia for 24 h, then exposed to X-rays (6 Gy). After 3 h, the cells were collected and incubated with Annexin V and propidium iodide for 15 min. The apoptotic cells were detected by flow cytometry using light scatter characteristics (BD Bioscience, Oxford, UK).

### Western blot analysis

Total cell lysates were prepared by harvesting cells in protein extraction buffer, and protein concentration was analyzed using the BCA protein assay kit. Equal amounts of proteins were separated on 6% or 10% SDS-polyacrylamide gel, transferred onto nitrocellulose membranes (Schleicher and Schuell Bio-Science). The membranes were blocked with 5% nonfat milk in TBST (Tris-buffered saline, pH 7.4 and 0.05% Tween 20) and incubated with HIF-1α antibody (1:200), VEGF antibody (1:250) and β-actin antibody (1:250) (all from Santa Cruz Biotechnology Inc, CA, USA) overnight at 4°C. The membranes were then incubated with alkaline phosphatase-conjugated goat anti-mouse IgG or goat anti-rabbit IgG (1:2000) for 1 h at room temperature, and the signals were detected by using enhanced chemiluminescence detection kit.

### Immunofuorescence

Cells in early log phase were trypsinized and plated in 24-well plates at a density of 4,000 cells/well. The cells were exposed to bererine and/or hypoxia for 24 h. Cells were fixed with methanol at −20°C for 20 min, and washed 3 times with PBS. Next the cells were incubated with goat anti-mouse HIF-1α antibody (1:250) or goat anti-rabbit VEGF antibody (1:250) for 16 h at 4°C, then incubated with anti-mouse-FITC or anti-rabbit-FITC (Jackson, USA) at a dilution of 1:150 for 1.5 h in the dark at room temperature. Finally, cells were mounted onto glass slides and observed under Confocal Laser Scanning Microscope (Zeiss LSM510).

### Tumor xenograft mouse models

Animal experiments were approved by Ethics Committee of Nanjing Medical University. Male BALB/C nude mice (4–5 weeks old) were provided by Nanjing Medical University Animal Center. Mice were then subcutaneously inoculated with LNCaP cells (5 × 10^6^ cells in 0.1 ml of PBS) at one site of the right armpit. When the average volume of tumour (visualized as small nodules at the sites of injection) increased to 150 mm^3^, the animals were randomly grouped into 6 different groups (n = 6): (1) vehicle (PBS), (2) 5 mg/kg berberine, (3) 10 mg/kg berberine, (4) 8 Gy IR, (5) 5 mg/kg berberine plus 8 Gy IR, (6) 10 mg/kg berberine plus 8 Gy IR. The mice in control group were treated with vehicle control, whereas the mice in 2, 3, 5 and 6 groups were given daily intraperitoneal injection of 5 or 10 mg/kg berberine every two days for 6 times. Tumors were irradiated by RS-2000 biological irradiator at a dose of 8 Gy with X-rays (2 Gy/min) delivered 2 h after injection on day 12. Tumor growth was measured every two days, the tumor volume was calculated according to the formula: Tumor volume = [length (L) × width (W)]^2^/2. The percentage of tumor growth inhibition (TGI) was calculated as follows: TGI (%) = [1-(mean change in the tumor volume in each group/mean change in the tumor volume in the control group)] × 100. The tumor doubling time (DT) was calculated as follows: DT = d × lg2/lg(V_d_/V_0_), where d was the length of time between two measurements, V_d_ was the volume of the tumor treated with X-ray and V_0_ was the volume of the tumor before the X-ray.

### Statistical analysis

Data were expressed as mean ± Standard Deviation (SD) of at least three independent experiments, and analyzed by SPSS statistical software system for Windows version 16.0 (SPSS Inc. Chicago, USA). Statistical significance was determined by using Student’s t-test and p < 0.05 was considered significant.

## Results

### Berberine sensitizes prostatic cancer cells to IR in normoxia and hypoxia conditions

**Figure 1:**
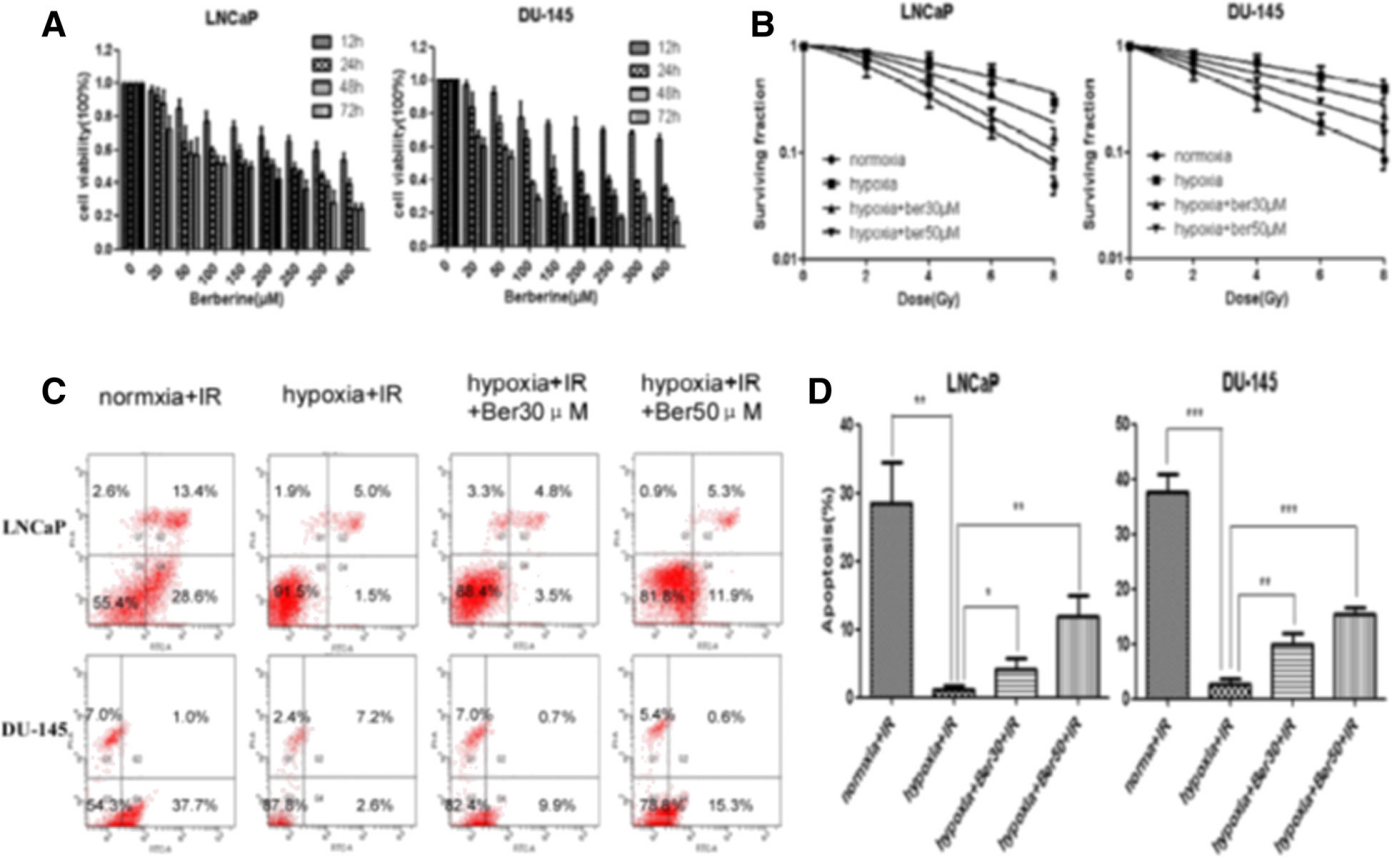
**Berberine sensitizes LNCaP and DU-145 cells to IR. (A)** berberine inhibited the growth of LNCaP and DU-145 cells in a time- and dose-dependent manner. **(B)** berberine inhibited the colony formation of LNCaP and DU-145 cells. **(C)** LNCaP and DU-145 cells were treated with berberine for 24 h in hypoxia, and then exposed to X-ray (6 Gy). After 3 h, apoptotic cells were detected by flow cytometry. **(D)** bars showing the rate of apoptosis from three independent experiments in LNCaP and DU-145 cells. LNCaP: p = 0.045, hypoxia + IR (6 Gy) vs. hypoxia + Ber30 + IR (6 Gy), DU-145: p = 0.0056, hypoxia + IR (6 Gy) vs. hypoxia + Ber30 + IR (6 Gy). Ber: berberine, 30: 30 μM, 50: 50 μM.

The viability of PCa cell lines LNCaP and DU-145 cells was detected after treatment with different concentrations of berberine for 12, 24, 48 and 72 h, respectively. MTT assay showed that berberine reduced the cell viability in a concentration and time dependent manner (Figure 1A). At 24 h, the IC50 of berberine for LNCaP and DU-145 cells were 220.36 μΜ and 163.56 μΜ, respectively. Thus we chose the concentrations of berberine at 30 and 50 μM for subsequent experiments.Clonogenic assay showed the radiation dose–response survival curves for LNCaP and DU-145 cells with or without berberine treatment for 24 h in normoxia and hypoxia conditions (Figure 1B). Both normoxic and hypoxic PCa cells that were pretreated with berberine exhibited a significantly increased sensitivity to IR, as reflected by the remarkable decrease in their ability to form colonies.The induction of apoptotic cell death is an important mechanism by which radio-/chemotherapy kills tumor cells. Next we detected apoptosis in LNCaP and DU-145 cells treated by berberine or/and IR (6 Gy). The rate of apoptotic cell death was higher in cells pretreated with 50 μM berberine than in cells pretreated with 30 μM berberine. Furthermore, berberine (30 μΜ and 50 μΜ) combined with IR, the rate of apoptotic cell death was significant higher in cells treated with both berberine and IR than in cells treated with IR alone (Figure 1C,D).

### Berberine inhibits HIF-1α and VEGF expression in prostatic cancer cells

**Figure 2:**
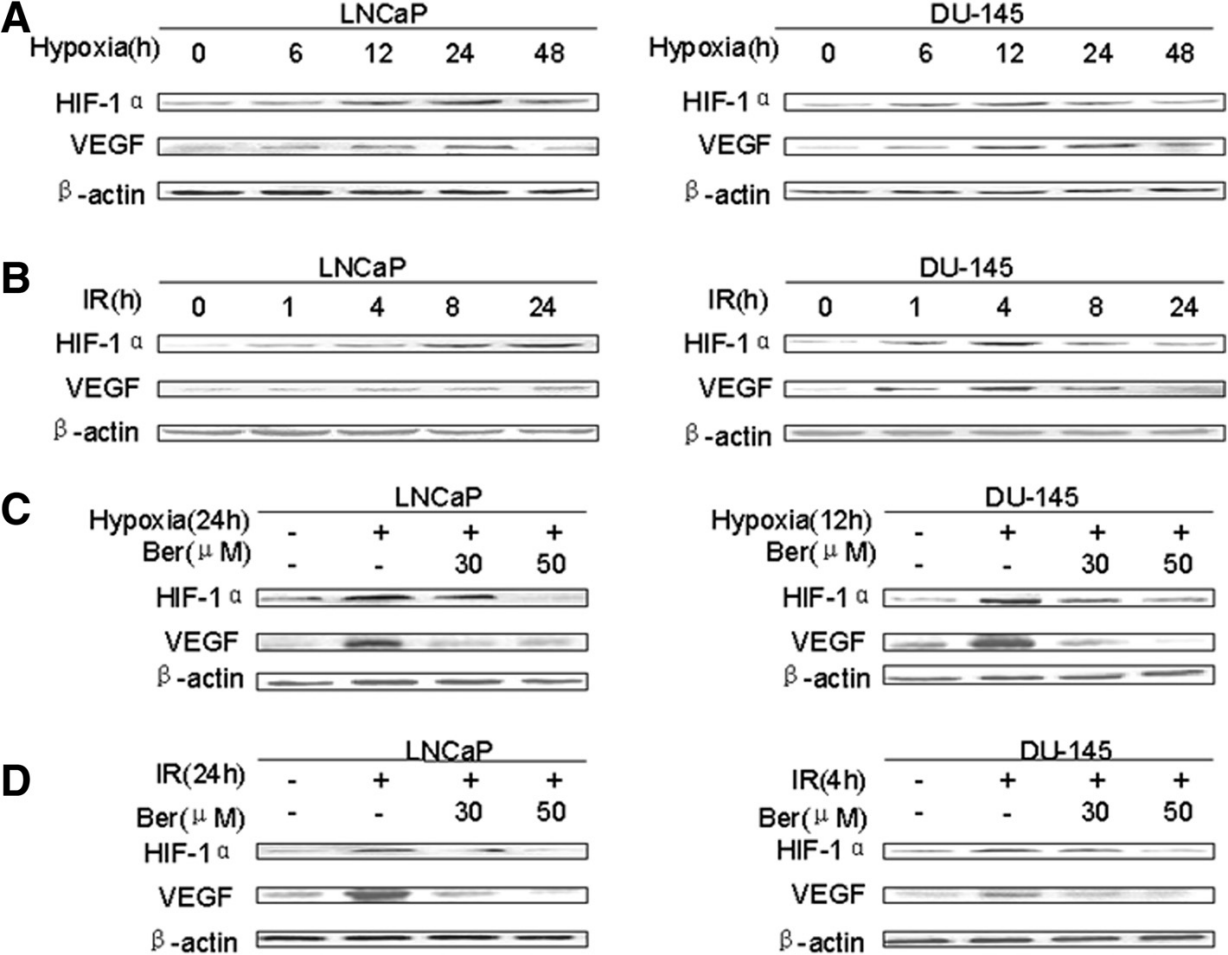
**Berberine inhibits the expression of HIF-1α and VEGF in LNCaP and DU-145 cells. (A)** hypoxia stimulated HIF-1α and VEGF expression in LNCaP and DU-145 cells. **(B)** IR stimulated HIF-1α and VEGF expression in LNCaP and DU-145 cells. **(C)** The expression of HIF-1α and VEGF was stimulated in hypoxia conditions and inhibited by berberine in LNCaP and DU-145 cells. **(D)** The expression of HIF-1α and VEGF was stimulated after IR and inhibited by berberine in LNCaP and DU-145 cells. β-actin was loading control. Ber: berberine.

**Figure 3:**
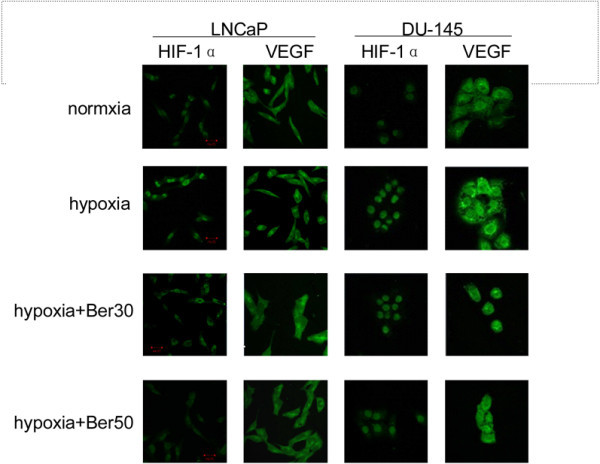
**Berberine inhibits the nuclear location of HIF-1α and VEGF in LNCaP and DU-145 cells.** Representative images of laser scanning confocal microscopy showed nuclear colocalization of HIF-1α and VEGF (green stain) in cells with hypoxia with or without berberine treatment (30 μM and 50 μM). Ber: berberine, 30: 30 μM, 50: 50 μM.

To investigate whether HIF-1α and VEGF are involved in berberine mediated radiosensitivity of prostatic cancer cells, we performed Western blot and immunofuorescence analysis to detect the protein expression of HIF-1α and VEGF. The results showed that hypoxia induced the expression of HIF-1α and VEGF in LNCaP and DU-145 cells (Figure 2A). Similarly, IR induced the expression of HIF-1α and VEGF in LNCaP and DU-145 cells (Figure 2B). However, the expression of HIF-1α and VEGF was decreased in hypoxic LNCaP and DU-145 cells treated with berberine compared to cells treated with hypoxia alone (Figure 2C). Furthermore, the expression of HIF-1α and VEGF was decreased in LNCaP and DU-145 cells treated with the combination of berberine and IR compared to cells treated with IR alone (Figure 2D). Moreover, the effects of berberine on HIF-1α and VEGF expression were concentration dependent (Figure 2C and Figure 2D).Furthermore, immunofluorescence analysis showed that HIF-1α was only located in the nucleus of normoxic cells, but VEGF was distributed in both the nucleus and cytoplasm (Figure 3). The translocation of HIF-1α and VEGF into the nucleus was observed in hypoxic LNCaP and DU-145 cells, which was inhibited prominently by berberine. And high concentration of berberine (50 μM) exhibited stronger inhibitory effects than the low concentration (30 μM). Taken together, these results suggest that berberine inhibits the expression of HIF-1α and VEGF in LNCaP and DU-145 cells.

### Berberine sensitizes prostatic cancer to IR in vivo

**Figure 4:**
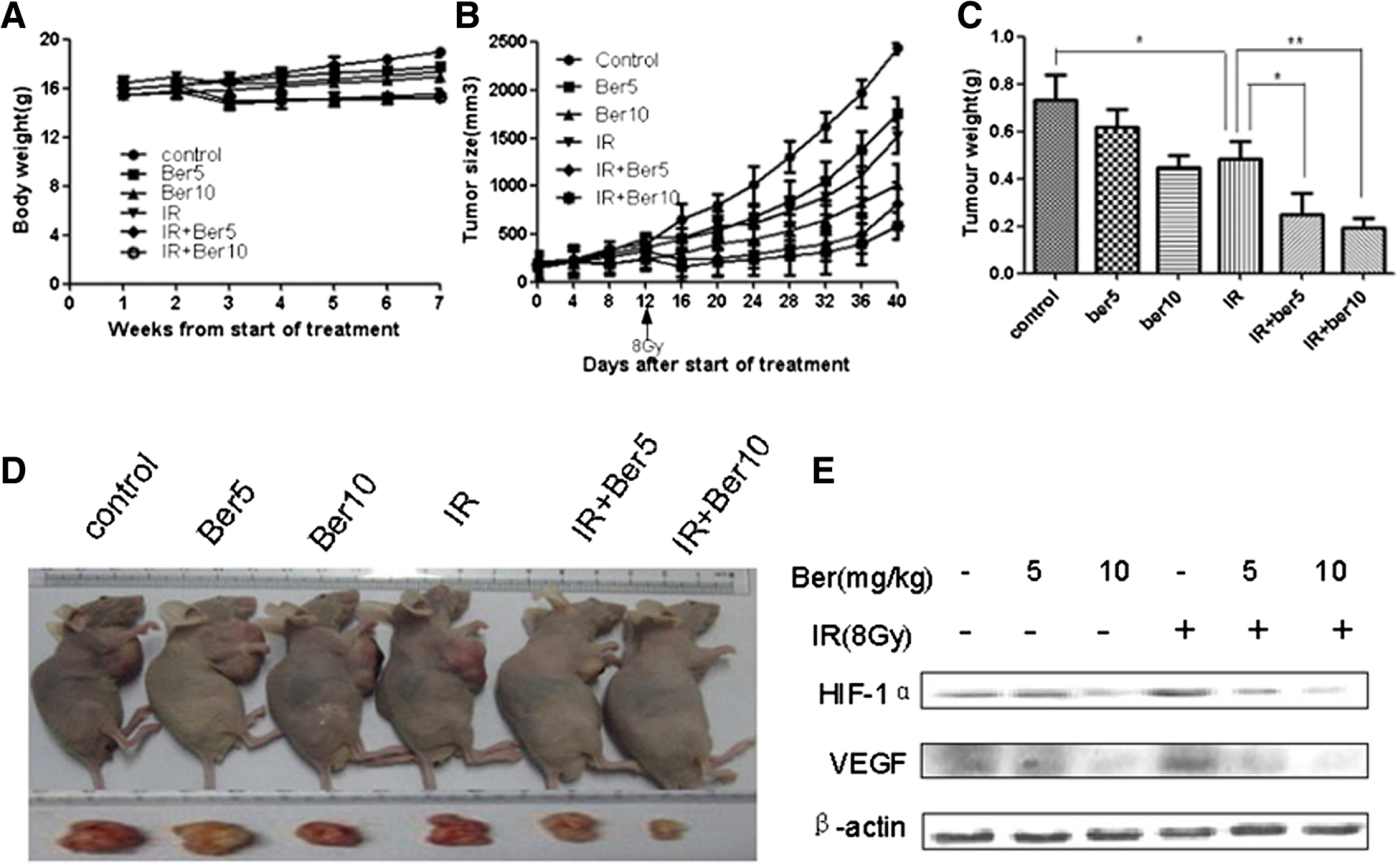
**Berberine increases radiosensitivity of LNCaP xenografts. (A)** Measurement of body weight in nude mice once per week. **(B)** Measurement of tumor volume in nude mice every four days. Data were presented as the relative tumor volume normalized to the initial tumor volume measured on day 0. **(C)** Measurement of tumor weight in the nude mice after sacrifice. P < 0.005, IR vs. berberine (5 or 10 mg/kg) + IR, **(D)** Visual observation of the tumors in each group. **(E)** Western blots showing the expression of HIF-1α and VEGF in LNCaP mouse xenograft tissues. Ber: berberine, 5: 5 mg/kg, 10: 10 mg/kg, IR: 8 Gy.

We next evaluated the effects of berberine on the radiosensitivity of PCa in vivo.Tumors were induced by s.c. injection of LNCaP cells into nude mice. We measured the body weight of the nude mice every week and the tumor volume every four days. The results showed that none of the treatment regimens produced any loss of body weight, which may be a sign of toxicity (Figure 4A). Combined treatment with IR and berberine significantly suppressed tumor volume and tumor weight in nude mice, compared to IR or berberine treatment alone (Figure 4B-D). As shown in Table 1, tumor growth delay (TGD) time for the 28-day treatment of Ber (5 or 10 mg/kg) alone was 12.3 or 13.1 days, not significantly different from that of control group (p = 0.3453, p = 0.2526, respectively). IR alone produced a TGD time of 5.7 days (p = 0.0116 compared to control group). The combined treatment with IR and berberine (5 or 10 mg/kg) resulted in a TGD time of 21.4 or 22.7 days, respectively (p < 0.05, compared with IR alone or berberine alone). Moreover, the combined treatment of IR and berberine (5 or 10 mg/kg) resulted in tumor growth inhibition of 66.6% and 75.9% on Day 40, respectively. Collectively, these data suggest that berberine sensitizes PCa to IR in vivo.

**Table 1:** Effect of berberine on response of ECA109 xenografted tumor to irradiation

**Treatment**	**n**	Inhibition (%)^*^	Doubling time (days)^**^	Absolute growth delay (days)^***^	**Normalized growth delay (days)**	**Enhancement factor**
Control	5		10.8 ± 1.2			
Ber5	5	27.6 ± 6.3	12.3 ± 2.5	1.5(12.3-10.8)		
Ber10	5	58.1 ± 8.6	13.1 ± 3.0	2.3(13.1-10.8)		
IR	5	37.8 ± 6.9	16.5 ± 1.5	5.7(16.5-10.8)		
IR + Ber5	5	66.6 ± 9.2	21.4 ± 0.7	10.6(21.4-10.8)	9.1(10.6-1.5)	1.6(9.1/5.7)
IR + Ber10	5	75.9 ± 5.9	22.7 ± 4.6	11.9(22.7-10.8)	9.6(11.9-2.3)	1.7(9.6/5.7)

*Tumor growth inhibition rate was calculated based on the tumor volume on Day 40.

^**^Absolute growth delay: The doubling tumor time of treatment group minus that of control group;

^***^Normalized growth delay: The time of absolute growth delay of tumor in IR combined with Ber group minus that of Ber group.

### Berberine inhibits HIF-1α and VEGF expression in xenografts induced by IR

Finally, we examined the effects of berberine on the expression of HIF-1α and VEGF in xenografts. Western blot analysis showed that berberine decreased IR-induced expression of HIF-1α and VEGF in LNCaP xenografts in a concentration dependent manner (Figure 4E).

## Discussion

It is crucially to predict pathological outcomes prior to surgery for appropriate surgical indication of PCa patients who receive radical prostatectomy [7]. Several biomarkers have been implicated in the initiation and progression of PCa [8,9]. Currently, the radiation resistance of prostate cancer remains the primary obstacle to improve patient survival. Several studies have investigated the mechanism underlying the radiosensitivity for the treatment of PCa. The inactivation of clusterin by anti-sense technology improved the outcomes of radiation therapy for prostate cancer patients [10]. Furthermore, a recent study showed that arsenic trioxide enhanced the radiation sensitivity of PCa through the inhibition of Akt/mTOR signaling pathways [11]. Importantly, HIF-1α is commonly upregulated in hypoxic tumour tissues, which may render resistance to radiotherapy [12]. Some anti-cancer drugs increase cancer cells radiosensitivity by downregulating HIF-1α [13].

It is well known that natural compounds confer radiosensitivity on cancer cells, such as curcumin and berberine [14-16]. However, there has been no report on the effects of berberine on the radiosensitivity of PCa. In the current study, we demonstrated for the first time that berberine could efficiently downregulate HIF-1α expression in hypoxic prostate cancer cells in vitro and in vivo. Berberine treatment also inhibited the upregulation of HIF-1α induced by IR in vitro and in vivo. Consistent with the downregulation of HIF-1α, we observed that berberine decreased colony formation and increased apoptosis in hypoxic LNCaP and DU-145 cells. Therefore, berberine increases the radiosensitivity of prostate cancer and better therapy efficacy could be achieved for combined treatment of PCa with IR and berberine.

## Conclusions

In summary, we showed that berberine at low concentrations substantially radiosensitized hypoxic prostate cancer cells by downregulating HIF-1α and VEGF expression, which may contribute to tumor aggressiveness, invasiveness and resistance to IR. Our findings suggest that berberine could be a novel radiosensitizer for PCa therapy.

## Competing interests

The authors declare that they have no competing interests.

## Authors’ contributions

QZ and JM wrote the manuscript. QZ, CZ, XY, BY, JW, YK, ZW, DL, GH, ZM, JC, GT, and SD performed the experiments on cell and molecular biology. WM performed statistical analysis. XS and JM supervised the study. All authors have read and approved the manuscript.
